# Alterations of sphingosine-1-phosphate and its receptors in type 1 diabetes mellitus: an integrated clinical and single-cell transcriptomic study

**DOI:** 10.3389/fimmu.2026.1838952

**Published:** 2026-06-08

**Authors:** Dongyan Chen, Fuhua Zhang, Tian Ren, Haiyan Chi, Mengyang Shen, Shiyu Kang, Xun Li, Peng Lin

**Affiliations:** 1 Department of Otolaryngology, Qilu Hospital of Shandong University, Jinan, Shandong, China; 2 Department of Endocrine and Metabolic Diseases, Qilu Hospital, Cheeloo College of Medicine, Shandong University, Jinan, China; 3 Key Laboratory of Endocrine and Metabolic Diseases, Shandong Province Medicine and Health, Jinan, Shandong, China; 4 Jinan Clinical Research Center for Endocrine and Metabolic Disease, Jinan, Shandong, China; 5 Internal Medicine Department, The First Rongjun Youfu Hospital of Shandong Province, Jinan, Shandong, China; 6 Department of Endocrinology, Weihai Municipal Hospital, Cheeloo College of Medicine, Shandong University, Weihai, Shandong, China

**Keywords:** immune cell atlas, inflammation, S1PR1, single-cell RNA sequencing, sphingosine-1-phosphate, type 1 diabetes mellitus

## Abstract

**Background:**

Type 1 diabetes (T1DM) is a T cell-mediated autoimmune disease. The sphingosine-1-phosphate (S1P) signaling pathway regulates lymphocyte trafficking, but its status in T1DM remains poorly characterized. This study investigated S1P and sphingosine-1-phosphate receptor 1(S1PR1) alterations in T1DM through integrated single-cell transcriptomic and clinical analysis.

**Main methods:**

We performed single-cell RNA sequencing (scRNA-seq)on colonic tissues from T1DM patients and healthy controls, complemented by analysis in non-obese diabetic (NOD) mice at pre-diabetic (4 weeks) and disease stages (12 weeks).Serum total S1P, HDL-bound S1P, and apolipoprotein M (ApoM) were quantified, and transcriptomic functional enrichment of intestinal T cell subsets was further performed.

**Results:**

scRNA-seq showed differences in S1P receptor expression in intestinal immune cells between T1DM patients and healthy controls. S1PR1 expression was lower in T cell subsets, particularly CD8+ effector T cells, in T1DM patients. In NOD mice, S1PR1 expression showed a clustered pattern at 4 weeks and was more diffuse with lower levels in lymphocytes at 12 weeks. Clinically, serum total S1P was significantly elevated in T1DM and independently increased T1DM risk, while high-density lipoprotein-bound (HDL-bound) sphingosine-1-phosphate (HDL-S1P) was decreased with unchanged apolipoprotein M (ApoM) levels. Serum S1P positively correlated with glycated hemoglobin (HbA1c) and interleukin-6 (IL-6), and was inversely associated with hypoglycemic episodes. Transcriptomic enrichment indicated functional reprogramming of intestinal CD8^+^ effector T and regulatory T cells.

**Conclusion:**

T1DM is associated with alterations in the S1P-S1PR1 pathway, characterized by reduced intestinal S1PR1 expression, elevated circulating total S1P, and decreased functional HDL-bound S1P despite stable ApoM.S1P is independently associated with T1DM status and correlates with glycemic control and inflammation, suggesting its potential as a disease-related biomarker.

## 1 Introduction

Type 1 diabetes mellitus (T1DM) is an organ-specific autoimmune disease (1) mediated by autoreactive T lymphocytes and characterized by the progressive destruction of pancreatic βcells (2). While the interplay between genetic susceptibility and environmental triggers has been extensively studied, the precise molecular mechanisms guiding autoreactive T cells to home to pancreatic islets remain incompletely understood. In recent years, increasing attention has been paid to the role of intestinal immune status in the pathogenesis of T1DM (3). As the largest immune organ and microbial reservoir (4), the intestinal tract may contribute to islet autoimmunity (3) by generating or activating specific immune cell subsets and facilitating their migration to pancreas (5, 6).

A central but unresolved question is how lymphocytes emigrate from the intestinal tissue to the distant pancreas. The signaling axis composed of sphingosine-1-phosphate (S1P) and its five G protein-coupled receptors (S1PR1–S1PR5) serves as the core navigation system regulating lymphocyte egress from lymphoid organs and barrier tissues (e.g., the gut) into the circulatory system (7, 8). Among them, S1PR1 is regarded as the principal receptor mediating lymphocyte extravasation (9). Notably, recent studies have provided direct evidence for the pivotal role of S1P in intestinal immunity (10, 11). Using *in vivo* imaging and single-cell technologies, a high-resolution “migration map” of intestinal immune cells revealed that all major lineages emigrate in an S1P-dependent manner, and after migration they retain molecular imprints of their intestinal origin and can home to distinct distal inflammatory or tumor sites (11). In CD4+ T cells, S1PR1 and S1PR4 cooperate with endothelial S1PR2 to finely regulate migration to afferent lymphatic vessels, indicating a precise regulatory mechanism of S1P signaling in the migration of tissue-specific immune cells (9, 12, 13).

At the therapeutic level, the S1P pathway has already been successfully targeted. S1P receptor modulators (such as fingolimod and ozanimod) antagonize S1PR1, thereby blocking lymphocyte egress from secondary lymphoid tissues, and are approved for multiple sclerosis and ulcerative colitis (10, 14). Their efficacy in ulcerative colitis directly demonstrates that regulating S1P-dependent migration can change the intestinal related immunopathological process (15–17).

Despite these advances, the role of S1P in the autoimmune β-cell destruction of T1DM remains poorly characterized. Studies on circulating S1P levels in T1DM have yielded inconsistent results (18–20), and more critically, whether the expression of S1P receptors on specific immune cell subsets—particularly pathogenic T cells—is altered in the intestinal microenvironment of T1DM patients at single-cell resolution has not been explored. Such alterations may influence S1P-directed migratory or tissue-retentive properties and are relevant for evaluating the pathway’s therapeutic potential.

In this study, we combined human colonic scRNA-seq, longitudinal NOD mouse model, and clinical cohort analysis to profile S1P/S1PR1 changes in T1DM. We characterized intestinal immune cell S1PR expression patterns, identified T cell subset-specific S1PR1 alteration, and analyzed clinical correlations of circulating S1P. We further investigated HDL-bound S1P and its carrier protein ApoM in T1DM patients. Transcriptomic enrichment revealed functional reprogramming of intestinal CD8Teff and Treg cells. This study provides descriptive molecular evidence for S1P/S1PR1 axis dysregulation and its potential involvement in intestinal immune homeostasis in T1DM.

## 2 Methods

### 2.1 Study population and ethics

This prospective cross-sectional study was approved by the Ethics Committees of Qilu Hospital of Shandong University and Weihai Municipal Hospital (Approval No: KYLL-2021-089). Written informed consent was obtained from all participants. Between November 2021 and December 2025, 100 patients meeting WHO/ADA diagnostic criteria for T1DM and 80 age- and sex-matched healthy controls (HC) were enrolled. Key exclusion criteria included other forms of diabetes, severe liver/kidney dysfunction, active malignancy, other systemic autoimmune diseases, recent use of immunosuppressants or S1PR modulators, and pregnancy. Supported by the General Program of the National Natural Science Foundation of China (No. 82370800, the only grant number).

### 2.2 Clinical data and sample collection

After an overnight fast, peripheral venous blood was collected into serum separator tubes. After clotting for 2 h at room temperature, samples were centrifuged at 4000 rpm for 20 min at 4 °C. Serum was aliquoted and stored at −80 °C until analysis. Clinical data including physical examination, laboratory tests (HbA1c, lipid profile, IL−6, etc.), and complications (hypoglycemic episodes, orthostatic hypotension, retinopathy, etc.) were recorded.

Colonic tissue samples were obtained from individuals undergoing colonoscopy for routine health screening, with tumor diseases excluded pathologically. All subjects provided written informed consent. The study was approved by the Ethics Committee of Qilu Hospital of Shandong University (KYLL-2020(KS)-572). Inclusion criteria: blood glucose level: fasting blood glucose ≥7.0mmol/L; Postprandial or random blood glucose ≥11.1mmol/L. The typical symptoms of three more and one less, namely hyperphagia, polydipsia, polyuria and weight loss, some patients directly manifest as symptoms of ketoacidosis such as dehydration, circulatory failure or coma. Low age of onset: onset in adolescents and younger age of onset. Impaired islet function: Islet function tests show a low level of insulin and C-peptide secretion with no obvious peak, and islet autoantibodies are usually positive. Some patients consented to genetic testing for the diagnosis of type 1 diabetes. T1DM is mainly diagnosed based on clinical manifestations, and insulin-dependent therapy caused by islet β cell destruction is the “gold standard” for the diagnosis of T1DM. At present, there is no exact diagnostic criteria for T1DM, and it is mainly diagnosed based on clinical features. Exclusion criteria: (1) type 2 diabetes or other special types of diabetes; (2) age < 18 or > 80 years old; (3) combined with active malignant tumor or previous history of malignant tumor; (4) complicated with other autoimmune diseases; (5) complicated with acute and chronic infections; (6) abnormal liver function (alanine aminotransferase or aspartate aminotransferase > 120U/L); Or renal insufficiency (eGFR < 60mL/min/1.73-); (7) recent use of immunosuppressive agents or S1P receptor modulators; (8) pregnancy status; (9) unqualified blood samples or missing clinical data.

Two 4−week−old and two 12−week−old female NOD mice were purchased from Jiangsu Huachuangxinuo Pharmaceutical Technology Co., Ltd. Mice were housed under specific pathogen−free conditions with a 12−h light/dark cycle and free access to food and water. All animal experiments were approved by the Animal Use Committee of Qilu Hospital (KYLL-2019(ks)-572, KYLL-2019-270).

### 2.3 Serum S1P, HDL-S1P and ApoM quantification

Serum S1P, HDL-S1P and ApoM concentrations were measured using a commercial human S1P enzyme-linked immunosorbent assay (ELISA)kit (Shanghai Enzyme-linked Biotechnology) following the manufacturer’s protocol. All samples were run in duplicate.

### 2.4 Single-cell RNA sequencing and bioinformatic analysis

Fresh human and mouse colon tissues were immediately placed in GEXSCOPE™ Tissue Preservation Solution (Singleron) and transported on ice to the Singleron laboratory. Tissues were washed with HBSS, minced into 2 mm fragments, and digested with GEXSCOPE™ Tissue Dissociation Solution at 37 °C for 15 min with agitation. The cell suspension was filtered through 40−μm strainers, centrifuged, and resuspended in PBS. Red blood cells were lysed with GEXSCOPE™ RBC Lysis Buffer. After washing, cell viability was assessed by trypan blue exclusion. Single−cell suspensions were loaded onto microfluidic devices, and scRNA−seq libraries were constructed using the GEXSCOPE™ Single−Cell RNA Library Kit following the manufacturer’s instructions. Sequencing was performed on an Illumina platform.

Raw data were processed using the Singleron pipeline. After quality control, gene expression matrices were analyzed using the Seurat package (v4.0) in R. Cells with <200 or >6000 detected genes, >20% mitochondrial reads, or <500 UMIs were excluded. Data were normalized using the LogNormalize method, and highly variable genes were identified. Principal component analysis was performed, and the first 20 principal components were used for UMAP dimensionality reduction and clustering. Cell types were annotated based on canonical markers. S1PR1–S1PR5 expression was visualized using FeaturePlot. Differential expression analysis between groups was performed using the Wilcoxon rank−sum test with an adjusted *P* < 0.05 and |log_2_FC| > 0.25 considered significant.

### 2.5 Gene set enrichment and functional enrichment analysis

Based on the differentially expressed genes (DEGs) screened from CD8^+^ effector T cells (CD8Teff) and regulatory T (Treg) cells of NOD mice, as well as intestinal T cells and CD8Teff cells from T1DM patients and healthy controls, we performed Gene Ontology (GO) and Kyoto Encyclopedia of Genes and Genomes (KEGG) enrichment analyses. Gene Set Enrichment Analysis (GSEA) was conducted to explore functional pathway alterations. Adjusted P < 0.05 and |log_2_FC| > 0.25 were set as the cutoff thresholds for significant enrichment. Bubble plots and heatmaps were used to visualize enriched pathways and representative DEG expression profiles.

### 2.6 Statistical analysis

Clinical data were analyzed using SPSS 27.0 and R (v4.2). Continuous variables are presented as mean ± SD and compared using Student’s t−test or Mann–Whitney U test, as appropriate. Categorical variables were compared by χ² test. Correlations were assessed by Pearson or Spearman correlation coefficients. Binary logistic regression was used to evaluate the association between S1P and T1DM status. Multivariable linear regression identified factors independently associated with S1P levels in T1DM patients. A two−sided *P* < 0.05 was considered statistically significant.

## 3 Results

### 3.1 Single-cell transcriptome analysis reveals differential expression patterns of S1P receptor family in intestinal immune cells

**Figure 1:**
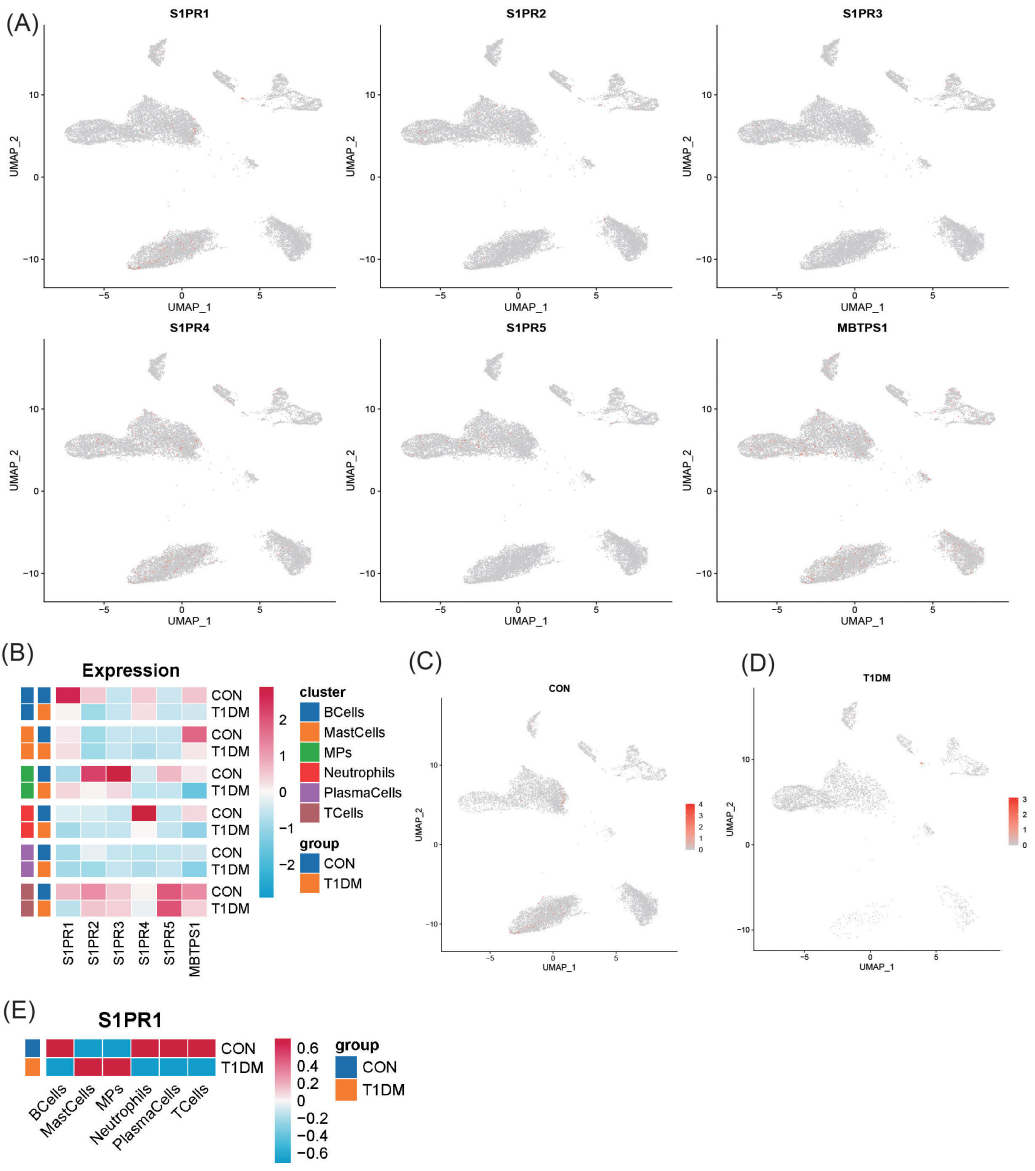
Single cell sequencing results-expression patterns of S1PS1PR in immune cells of CON and T1DM groups: **(A)** UMAP plots showing expression of S1PR1–S1PR5 across all colonic immune cells from healthy controls (CON) and T1DM patients. **(B)** Heatmap depicting average expression (color intensity) of cells expressing each S1P receptor in major immune cell types for CON and T1DM groups. **(C, D)** Feature plot highlighting S1PR1 expression distribution within the immune cell landscape. **(E)** Quantitative comparison of S1PR1 expression differences between CON and T1DM across immune cell types. Data are representative of n = 2 biological replicates in each group.

To characterize the expression of S1P receptors (S1PR1–S1PR5) in the intestinal immune microenvironment, we analyzed single-cell transcriptomic data from healthy controls (CON) and individuals with type 1 diabetes (T1DM). Visualization of the data showed distinct lineage-specific expression patterns: S1PR1 and S1PR5 were broadly distributed with partial overlap across immune cell types; S1PR3 displayed more restricted expression; and S1PR2 and S1PR4 were predominantly confined to specific subsets (**Figure 1A**).

Comparison between T1DM and CON groups revealed differences in the expression profiles of S1P receptors across multiple immune cell populations (**Figure 1B**). In T1DM samples, S1PR1 expression was higher in myeloid cells such as macrophages and in mast cells, whereas lower expression was observed in T cells. Further mapping of S1PR1 expression within intestinal immune populations confirmed its presence in specific subsets (**Figures 1C, D**). Quantification of S1PR1 expression differences across immune cell types between CON and T1DM groups substantiated a significant shift in the S1P receptor landscape within the colonic immune microenvironment of T1DM patients (**Figure 1E**).

### 3.2 T cell subset–specific S1PR1 downregulation in T1DM

**Figure 2:**
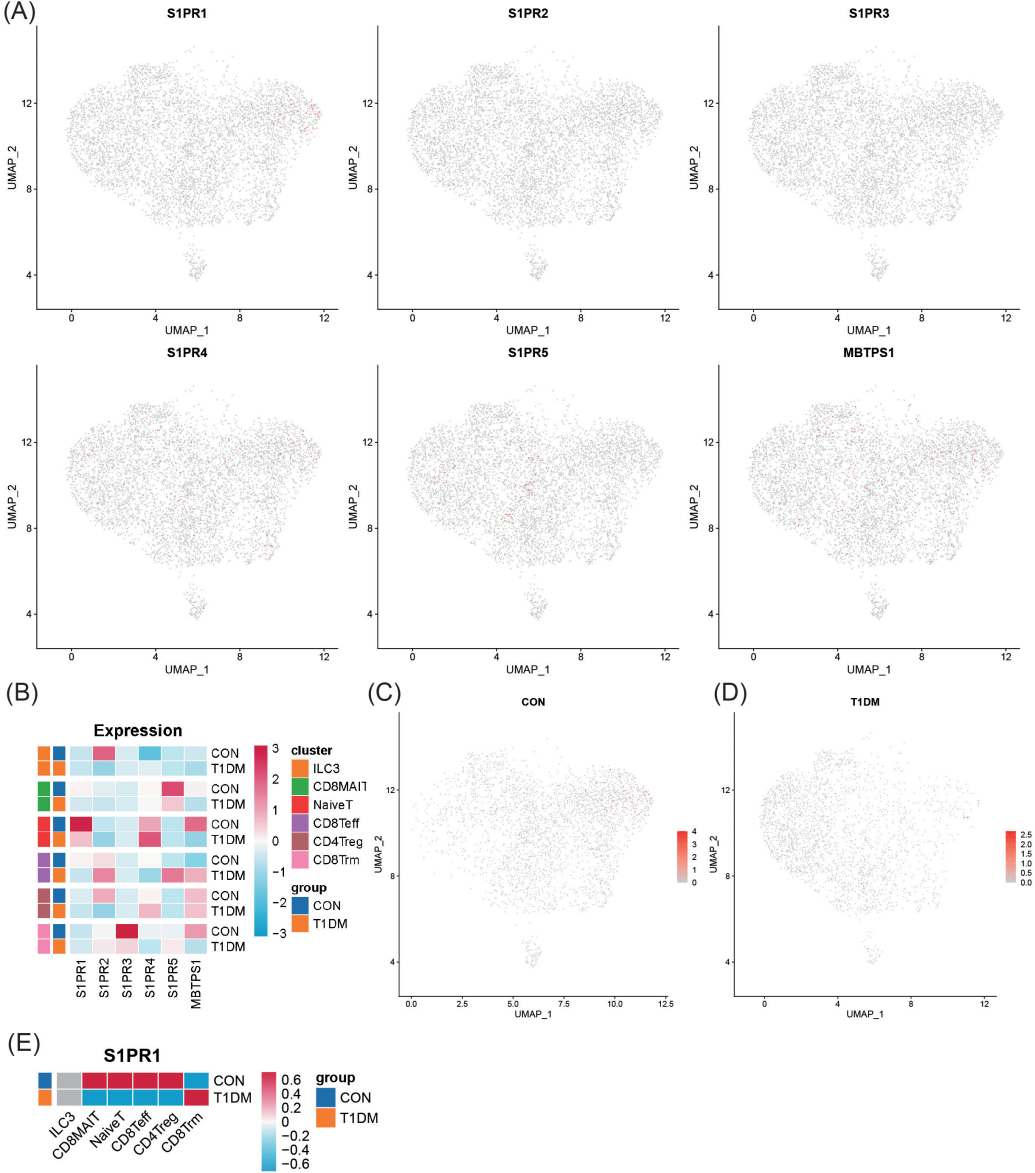
Single cell sequencing results-expression patterns of S1P/S1PR in T cells of CON and T1DM groups: **(A)** UMAP plots showing expression of S1PR1–S1PR5 across all colonic T cell subsets from healthy controls (CON) and T1DM patients. **(B)** Heatmap depicting average expression (color intensity) of cells expressing each S1P receptor in major T cell types for CON and T1DM groups. **(C, D)** Feature plot highlighting S1PR1 expression distribution within the T cell landscape. **(E)** Quantitative comparison of S1PR1 expression differences between CON and T1DM across T cell types. Data are representative of n = 2 biological replicates in each group.

To examine S1P receptor expression in adaptive immune cells, we focused on T cell subsets. Using high-resolution transcriptomic data, intestinal T cells were stratified into functionally distinct subsets, and the expression of S1PR1–S1PR5 and the regulatory factor MBTPS1 was profiled. UMAP visualization showed subset-specific distribution of S1P pathway components: S1PR1 was detected predominantly in naïve T cells and CD8+ effector T cells (CD8 Teff); S1PR4 and S1PR5 were primarily observed in CD8+ tissue-resident memory T cells (CD8 Trm) and CD4+ regulatory T cells (CD4 Treg); S1PR2 and S1PR3 were more broadly distributed with varying intensities across subsets (**Figure 2A**). MBTPS1 expression showed a distribution pattern similar to that of S1PR1, particularly in naïve T and CD8 Teff cells (**Figure 2B**).

Comparison of T1DM and healthy control samples showed lower S1PR1 expression intensity in the T1DM group, with the most pronounced differences in CD8 Teff and other effector subsets (**Figures 2C, D**). Quantitative analysis confirmed significantly lower S1PR1 expression in CD8 Teff cells from T1DM patients relative to controls, while S1PR1 expression in Treg cells showed considerable inter-individual variability (**Figure 2E**).

### 3.3 Dynamic spatiotemporal remodeling of S1PR1 in the NOD mouse model

**Figure 3:**
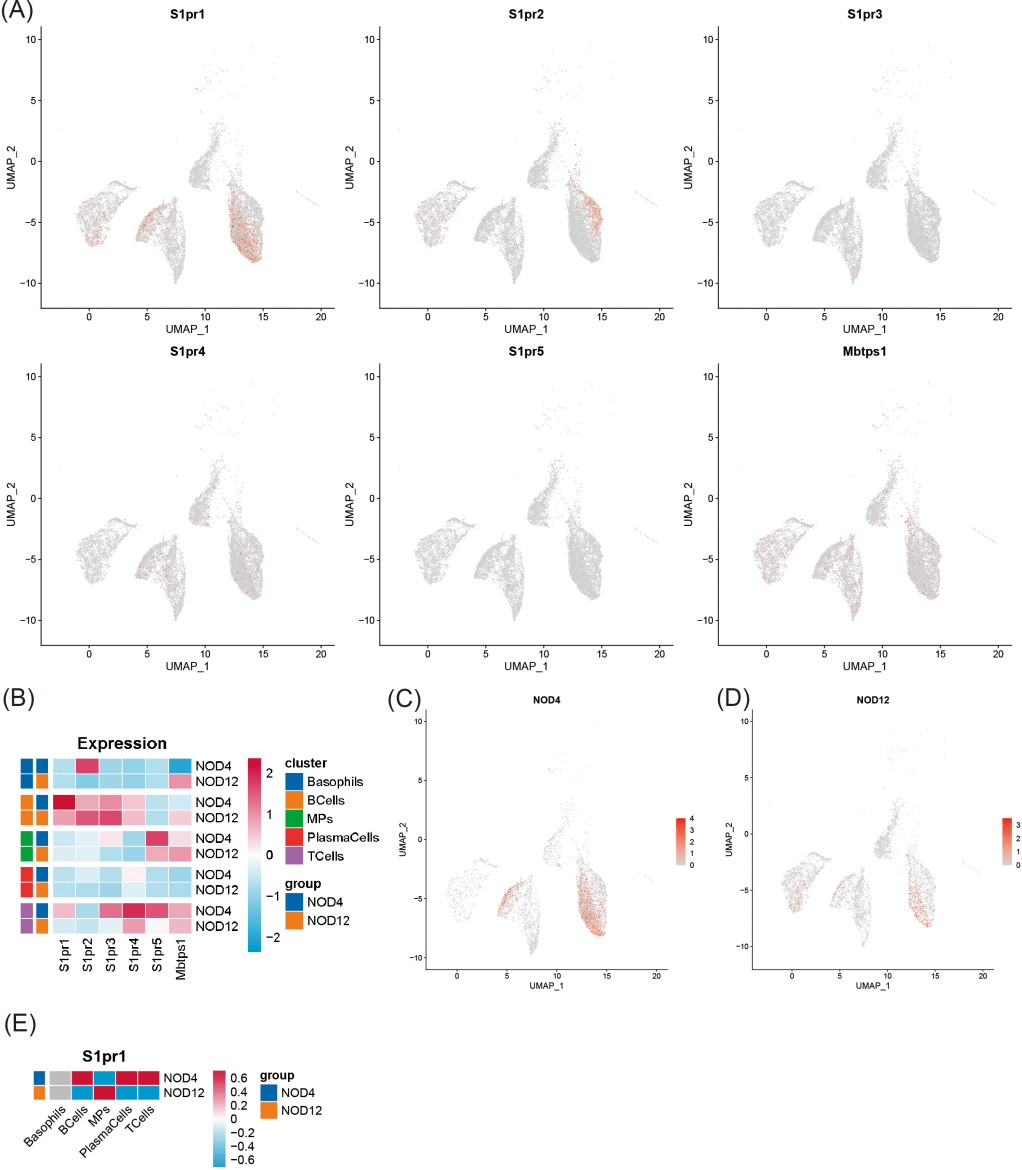
Distribution of S1P receptor expression in intestinal immune cells of 4 and 12-week-old NOD mice: **(A)** UMAP plots showing expression of S1PR1–S1PR5 across colonic immune cells from NOD mice at pre-diabetic (4 weeks) and diabetic stage (12 weeks). **(B)** Heatmap comparing S1P receptor expression across immune cell types from NOD mice between 4 and 12 weeks. **(C, D)** Feature plot highlighting S1PR1 expression distribution within the immune cell landscape from NOD mice. **(E)** Quantitative analysis of S1PR1 expression changes across immune cell types from 4 and 12 weeks. Data are representative of n = 2 biological replicates in each group.

To assess changes in S1P receptor expression during disease progression, we analyzed colonic immune cells from NOD mice at 4 weeks (pre-diabetic stage) and 12 weeks of age (diabetes-prone stage). UMAP projection showed subset-specific expression patterns at both time points: at 4 weeks, S1PR1 was detected predominantly in T cells, B cells, and plasma cells; S1PR2 and S1PR4 were largely confined to myeloid subsets such as macrophages and dendritic cells; S1PR3 and S1PR5 were observed in both lymphoid and myeloid compartments (**Figure 3A**). Comparison between the two time points revealed changes in the expression levels of multiple receptors across cell types (**Figure 3B**).

For S1PR1, differences in both spatial distribution and expression intensity were noted between the two stages. At 4 weeks, S1PR1 expression was concentrated in lymphocytes; by 12 weeks, its expression was more broadly distributed, with detection in myeloid cells (**Figures 3C, D**). Quantitative analysis showed lower S1PR1 expression in T cells, B cells, and plasma cells at 12 weeks compared to 4 weeks, accompanied by higher expression in the macrophage/monocyte lineage (**Figure 3E**).

### 3.4 T cell subset–specific dynamics of S1PR1 expression

**Figure 4:**
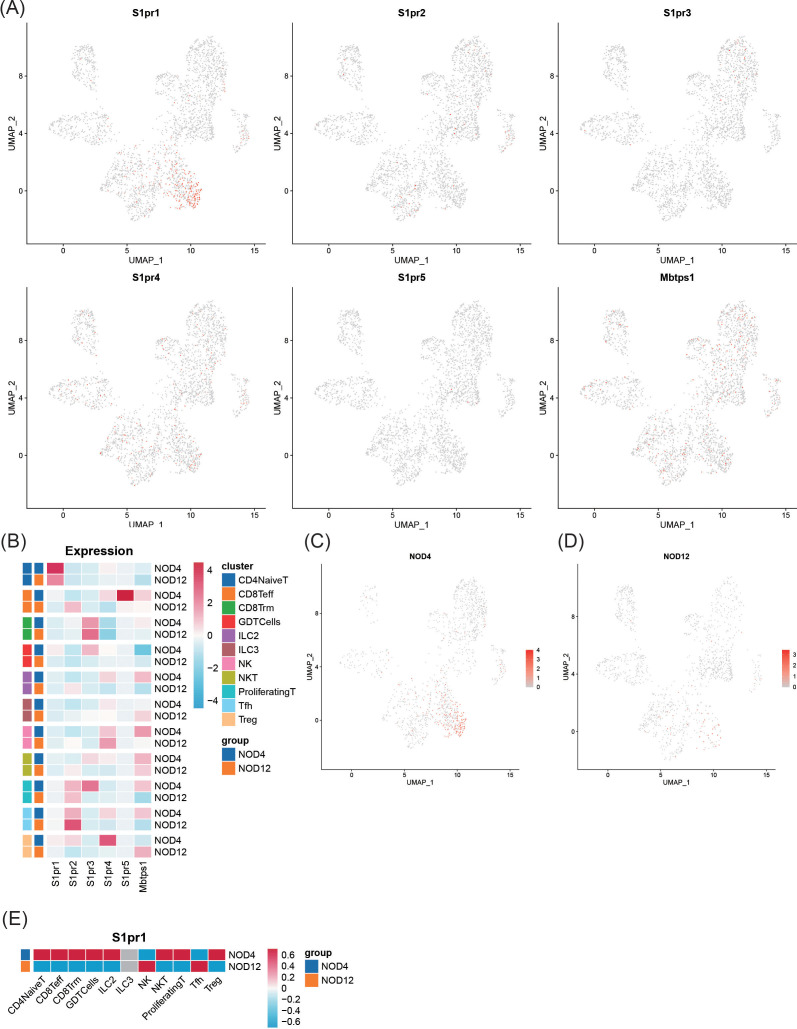
Distribution of S1P receptor expression in T cell subsets of intestine in 4 and 12-week-old NOD mice: **(A)** UMAP plots showing expression of S1PR1–S1PR5 across colonic T cells from NOD mice at pre-diabetic (4 weeks) and diabetic stage (12 weeks). **(B)** Heatmap comparing S1P receptor expression across T cell types from NOD mice between 4 and 12 weeks. **(C, D)** Feature plot highlighting S1PR1 expression distribution within the T cell landscape from NOD mice. **(E)** Quantification of S1pr1 expression changes in specific T cell subsets from 4 and 12 weeks. Data are representative of n = 2 biological replicates in each group.

To further examine S1P receptor expression within T cell subsets over time, we performed subset-specific single-cell analysis. Heatmap visualization showed the expression profiles of S1PR1–S1PR5 across colonic T cell subsets at both time points. S1PR1 was detected in CD4+ naïve T cells and CD8+ effector T cells; S1PR4 and S1PR5 were observed in CD8+ tissue-resident memory T cells and CD4+ regulatory T cells; S1PR2 and S1PR3 showed broader distribution with variable intensities across subsets (**Figure 4A**). These patterns were comparable to those observed in human T cells (**Figure 2A**).

Comparison of expression profiles between 4-week and 12-week time points showed differences in S1P receptor expression levels (**Figure 4B**). At 12 weeks, S1PR1 expression was lower in CD4+ naïve T cells compared to 4 weeks, with a similar trend observed in multiple T cell subsets, including CD8+ regulatory T cells (**Figures 4C, D**). The clustered expression pattern of S1PR1 at 4 weeks was less pronounced by 12 weeks (**Figures 4C, D**). This reduction in S1pr1 expression across T cell subsets was further confirmed by the aggregated expression profile, showing consistent downregulation from NOD4 to NOD12 mice (**Figure 4E**).

### 3.5 Functional enrichment analysis of intestinal T cell subsets

We further performed Gene Ontology (GO) and Kyoto Encyclopedia of Genes and Genomes (KEGG)enrichment analyses on intestinal T cells and CD8Teff cells from T1DM patients and healthy controls, as well as Treg and CD8Teff cells from NOD mice at 4 and 12 weeks of age (**Supplementary Figures 1**–**4**).

In human intestinal T cells, genes with altered expression were mainly enriched in TCR signaling, antigen processing and presentation, cytotoxicity, MAPK and IL-17 inflammatory pathways. In human CD8Teff cells, pathways related to apoptosis regulation, cellular stress and protein folding were significantly changed, indicating functional remodeling of effector and stress responses in T1DM-derived CD8Teff cells.

In NOD mice, intestinal CD8Teff cells at the disease stage (12 weeks) showed significant enrichment in type 1 diabetes mellitus, GPCR/chemokine receptor signaling, granzyme-mediated cytotoxicity and cell migration-related pathways. Ribosome biogenesis and translational pathways were also upregulated, suggesting enhanced activation status of CD8Teff during disease progression. Intestinal Treg cells from NOD mice also displayed alterations in protein folding, membrane microdomain and inflammatory signaling pathways, implying potential impairment of immune homeostasis.

These transcriptomic alterations indicated that intestinal T cell subsets in both human T1DM and NOD mice underwent transcriptional remodeling in GPCR/chemokine signaling, cellular stress and cytotoxic programs. These pathway changes are closely associated with the regulatory machinery of lymphocyte migration and activation, providing descriptive molecular clues for intestinal immune remodeling during T1DM progression, while direct functional verification requires further investigation.

### 3.6 Clinical characteristics and serum S1P levels in the study cohort

#### 3.6.1 Baseline characteristics

The baseline clinical characteristics of the 180 participants are presented in **Table 1**. The proportion of males was comparable between groups (HC: 68.8% vs. T1DM: 53.0%, *P* = 0.32). T1DM patients were slightly older than healthy controls (50.9 ± 16.4 vs. 46.0 ± 13.2 years, *P* = 0.026), with a mean difference of 4.9 years (95% CI 0.5–9.3 years). The prevalence of current smokers was similar (22.5% vs. 14.0%, *P* = 0.14), while current drinkers were fewer in the T1DM group (6.0% vs. 18.8%, *P* = 0.01), likely reflecting clinical advice. Hypertension prevalence did not differ significantly (33.8% vs. 26.0%, *P* = 0.26). Lipid profiles, including triglycerides (1.38 ± 0.72 vs. 1.40 ± 1.17 mmol/L, *P*= 0.935), total cholesterol (4.58 ± 1.06 vs. 4.71 ± 1.40 mmol/L, *P*= 0.475), and LDL−C (2.77 ± 0.88 vs. 2.52 ± 0.95 mmol/L, *P* = 0.078), were well balanced between groups, indicating that any observed differences in S1P are unlikely attributable to dyslipidemia.

**Table 1:** Baseline clinical characteristics of the healthy control and type 1 diabetes groups.

Variables	HC (n=80)	T1DM (n=100)	P Value
Male gender, n (%)	55 (68.8)	53 (53.0)	0.32
Smoker, n (%)	18 (22.5)	14 (14.0)	0.14
Drinker, n (%)	15 (18.8)	6 (6.0)	0.01
Hypertension, n (%)	27 (33.8)	26 (26.0)	0.26
Age (years)	46 ± 13.19	50.94 ± 16.37	0.026
TG (mmol/L)	1.38 ± 0.72	1.40 ± 1.17	0.935
TC (mmol/L)	4.58 ± 1.06	4.71 ± 1.40	0.475
LDL-C (mmol/L)	2.77 ± 0.88	2.52 ± 0.95	0.078

Data are mean ± SD unless stated. HC, healthy control; T1DM, type 1 diabetes mellitus; TG, triglycerides; TC, total cholesterol; LDL-C, low-density-lipoprotein cholesterol; S1P, sphingosine-1-phosphate.

#### 3.6.2 Comparison of S1P concentrations

**Figure 5:**
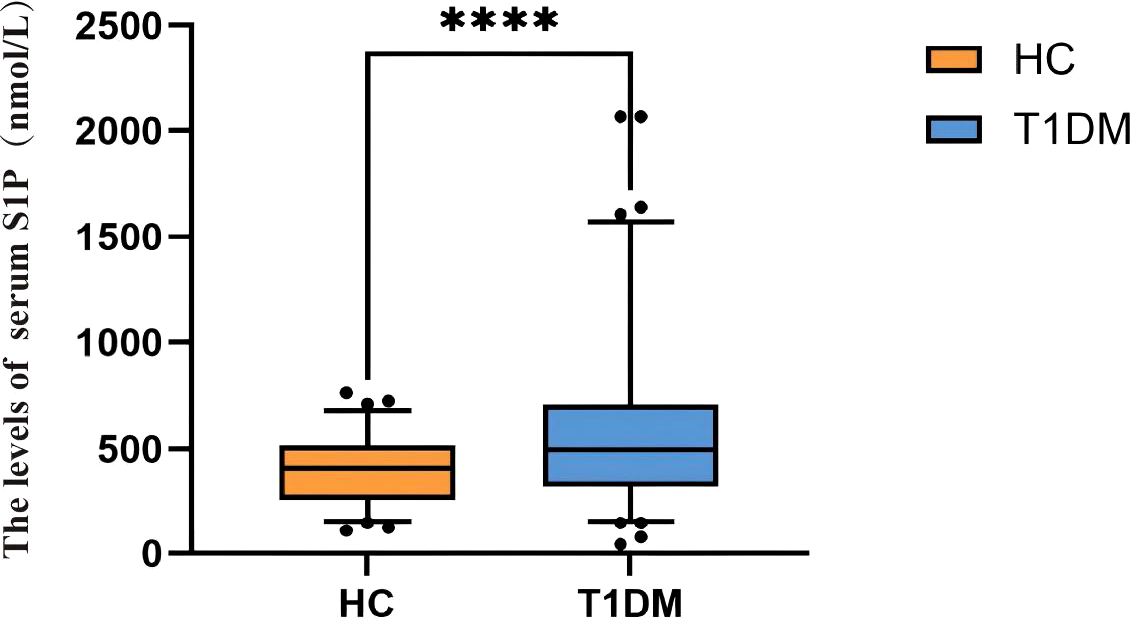
Box plot of serum S1P levels between the two group: Box plot comparing serum S1P concentrations (nmol/L) between healthy controls (HC, n=80) and T1DM patients (n=100). Central line, median; box limits, interquartile range (IQR); whiskers, 1.5× IQR; dots, outliers. ****p < 0.0001.

Serum S1P levels were markedly elevated in T1DM patients compared to healthy controls (871.6 ± 771.8 vs. 457.5 ± 253.9 nmol/L, *P* < 0.001; **Table 2**), with a mean difference of 414.1 nmol/L (95% CI 241–587 nmol/L). The effect size (Cohen’s d ≈ 0.78) indicates a large and clinically relevant difference. The T1DM group exhibited greater inter−individual variability (coefficient of variation: 88.5% vs. 55.5%), confirmed by Levene’s test for heterogeneity of variance (F ≈ 23.4, *P* < 0.001). After excluding outliers exceeding 3 standard deviations (14 T1DM samples with S1P > 2315.3 nmol/L and 9 HC samples with S1P > 761.8 nmol/L), the difference remained highly significant (606.7 ± 431.8 vs. 390.8 ± 160.5 nmol/L, *P*< 0.001; **Table 3**), confirming that the elevation was not driven by extreme values. The box plot visually confirms the marked elevation and greater dispersion in the T1DM group (**Figure 5**).

**Table 2:** Comparison of S1P concentrations between the healthy control and type 1 diabetes groups.

Group	n	S1P (nmol/L)	P
HC	80	457.50 ± 253.94	
T1DM	100	871.60 ± 771.75	<0.01***

*** indicates p < 0.001. Represent statistically significant differences between the compared groups.

**Table 3:** Comparison of S1P concentrations after outlier removal in both groups.

Group	n	S1P (nmol/L)	P
HC	71	390.81 ± 160.48	
T1DM	86	606.70 ± 431.83	<0.01***

*** indicates p < 0.001. Represent statistically significant differences between the compared groups.

#### 3.6.3 S1P is independently associated with T1DM risk

Binary logistic regression demonstrated that serum S1P concentration was a significant predictor of T1DM status (B = 0.002, *P* < 0.001; **Table 4**). Each 0.01 nmol/L increment in S1P was associated with a 0.2% increase in the odds of having T1DM (OR = 1.002, 95% CI 1.001–1.003). This translates to a clinically meaningful 22% increase in odds per 100 nmol/L S1P elevation (OR ≈ 1.22, 95% CI 1.14–1.31). The lower bound of the confidence interval remaining above 1.0 confirms the stability and significance of this association.

**Table 4:** Binary logistic regression analysis of T1DM risk factors.

Variable	B	OR (95%CI)	P value
S1P	0.002	1.002	<0.001
Constant	-0.725	0.484	<0.001

*** indicates p < 0.001. Represent statistically significant differences between the compared groups.

#### 3.6.4 Correlation between S1P concentration and clinical complications in the T1DM subgroup

Multivariable linear regression analysis within the T1DM group, with serum S1P as the dependent variable, revealed significant positive correlations with interleukin-6 (B = 40.46, 95% CI 8.57–72.34, *P* = 0.01) and glycated hemoglobin (B = 53.58, 95% CI 8.42–98.73, *P* = 0.02) (**Table 5**). No significant associations were observed with age, sex, smoking, alcohol consumption, hypertension, hyperlipidemia, BMI, hs-CRP, complement C1q, fasting C-peptide, GADA, IAA, fasting glucose, UACR, or urinary microalbumin (all *P*> 0.05).

**Table 5:** Linear regression analysis of risk factors of T1DM.

		B (95%CI)	SE	t	P
constant		1432.64 (-324.25,3189.54)	882.12	1.62	0.11
Age (year)		-5.00 (-18.27,8.27)	6.66	-0.75	0.46
Gender		102.45 (-260.54,465.43)	182.25	0.56	0.58
Smoker		-180.76 (-841.21,479.68)	331.60	-0.55	0.59
Drinker		-119.62 (-1076.63,837.38)	480.50	-0.25	0.80
Hypertension		135.20 (-288.53,558.94)	212.76	0.64	0.53
Hyperlipemia		49.34 (-385.35,484.02)	218.25	0.23	0.82
Years of schooling (year)		-40.53 (-82.26,1.21)	20.96	-1.93	0.06
Disease course of diabetes (month)		-0.37 (-2.45,1.72)	1.05	-0.35	0.73
TG (mmol/L)		-52.95 (-207.19,101.29)	77.44	-0.68	0.50
TC (mmol/L)		72.45 (-193.61,338.50)	133.59	0.54	0.59
LDL-C (mmol/L)		-105.37 (-462.57,251.84)	179.35	-0.59	0.56
BMI (kg/m^2^)		-21.25 (-69.85,27.35)	24.4	-0.87	0.39
hs-CRP (mg/L)		-98.94 (-265.98,68.09)	83.87	-1.18	0.24
IL-6 (pg/mL)		40.46 (8.57,72.34)	16.01	2.53	0.01***
Complement C1q (ug/mL)		3.62 (-48.51,55.74)	26.17	0.14	0.89
Fasting C-peptide (mg/mL)	-105.60 (-281.26,70.06)		88.2	-1.2	0.24
GADA (U/mL)	-0.27 (-1.45,0.09)		0.59	-0.47	0.64
IAA (U/mL)	-1.42 (-4.76,1.93)		1.68	-0.84	0.4
Fasting blood-glucose (mmol/L)	-3.43 (-25.35,18.49)		11.01	-0.31	0.76
HbA1c (%)	53.58 (8.42,98.73)		22.67	2.36	0.02***
UACR (mg/g)	-0.21 (-1.80,1.38)		0.8	-0.26	0.79
Microalbuminuria in urine (mg/L)	-0.08 (-0.93,0.78)		0.43	-0.18	0.86

*** indicates p < 0.001. Represent statistically significant differences between the compared groups.

#### 3.6.5 S1P and clinical complications

Univariate logistic regression analysis showed that higher serum S1P levels were inversely associated with hypoglycemic episodes within the past 12 months (OR = 0.999, 95% CI 0.999–1.000, *P* = 0.034). No significant correlations were observed for orthostatic hypotension, ischemic heart disease, retinopathy, chronic hypoglycemia, or ketosis (all *P* > 0.05; **Table 6**). Ordinal regression analysis demonstrated no significant associations between serum S1P and neuropsychological indicators including MoCA, MMSE, HAMD-17, and PSQI scores (all *P* > 0.05; **Table 7**).

**Table 6:** Regression analysis of related complications.

Complication	OR (95%CI)	P
OH	0.999 (0.998,1.000)	0.070
Ischemic heart disease	0.999 (0.998,1.001)	0.252
Fundus lesions	1.000 (0.999,1.001)	0.644
Hypoglycemia episodes within the past 12 months	0.999 (0.999,1.000)	0.034***
Chronic episodes of hypoglycemia	1.001 (1.000,1.001)	0.245
Ketosis	1.000 (1.000,1.001)	0.432

*** indicates p < 0.001. Represent statistically significant differences between the compared groups.

**Table 7:** Analysis of the relationship between the level and the indicators related to the nerve.

	SE	Wald χ^2^	P	OR (95%CI)
Moca	<0.001	1.202	0.273	1.000 (0.999 -1.000)
MMSE	<0.001	0.478	0.489	1.000 (0.999 - 1.000)
HAMD-17	<0.001	0.264	0.067	1.000 (0.999 - 1.001)
PSQI	<0.001	0.000	0.984	1.000 (0.999 - 1.000)

#### 3.6.6 HDL-S1P and ApoM alterations in T1DM

**Figure 6:**
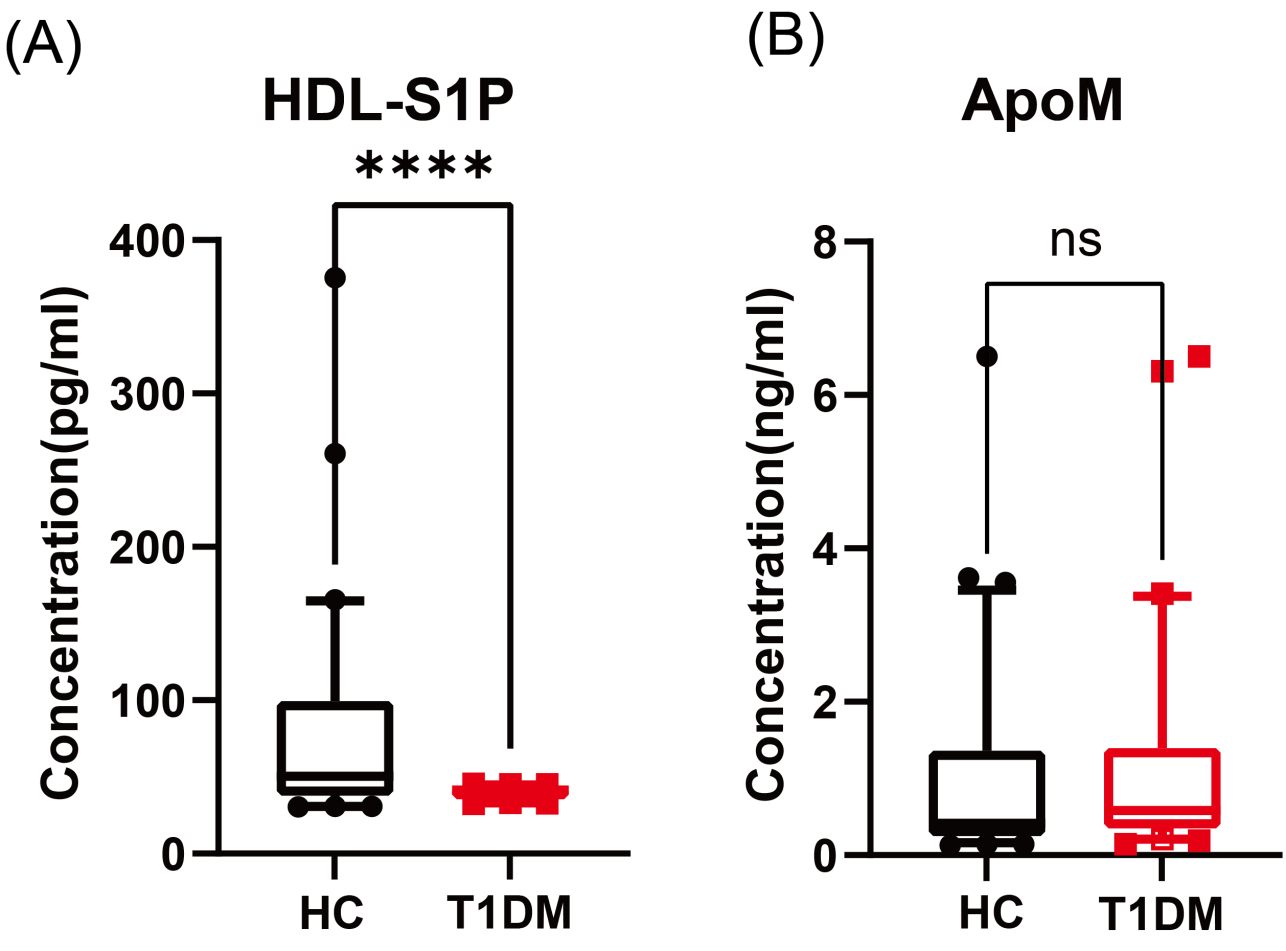
Box plot of serum HDL-bound S1P and ApoM levels in T1DM patients and healthy controls. Comparing serum HDL-S1P and ApoM concentrations between healthy controls (HC, n=70) and T1DM patients (n=70). Central line, median; box limits, interquartile range (IQR); whiskers, 1.5× IQR; dots, outliers. ****p < 0.0001, ns, not significant.

Given the critical role of HDL-bound S1P (HDL-S1P) as the biologically active form of circulating S1P, we further measured serum HDL-S1P and its major carrier protein ApoM. Compared with healthy controls, T1DM patients showed significantly reduced HDL-S1P levels (*P* < 0.0001), while serum ApoM concentration remained unchanged (ns, **Figure 6**). These results indicate that the elevated total serum S1P in T1DM is accompanied by a reduction in functional HDL-bound S1P, despite stable ApoM protein abundance.

## 4 Discussion

This study examined the S1P-S1PR1 pathway in T1DM using human single-cell transcriptomics, a longitudinal NOD mouse model, and a clinical cohort. The main observations were: (1) S1PR1 expression was lower in intestinal T cells of T1DM patients, particularly in CD8+ effector T cell subsets, compared to controls; (2) In NOD mice, S1PR1 expression showed a clustered pattern in lymphocytes at 4 weeks and a more diffuse pattern with lower levels in lymphocytes at 12 weeks; (3) Serum circulating total S1P levels were higher in T1DM patients and showed positive correlations with IL-6 and HbA1c; (4) Further detection revealed decreased serum HDL-bound S1P while ApoM levels remained unchanged in T1DM patients.

### 4.1 Correlation between circulating S1P level rise and its functional state

In this study, the serum total S1P level of patients with T1DM increased significantly, but at the same time, the expression of its main receptor S1PR1 on intestinal T cells decreased. Combined with our additional detection, we further found that HDL-bound S1P was significantly decreased in T1DM, whereas serum ApoM concentration remained unaltered. This indicates that elevated total S1P cannot simply represent enhanced biological activity, and there exists an obvious functional dissociation between circulating total S1P and HDL-bound functional S1P in T1DM. Recent studies have highlighted that total S1P measurement alone may not reflect biologically active S1P, as its function is critically dependent on carrier molecules—primarily high-density lipoprotein (HDL) via apolipoprotein M (ApoM) and albumin (21–23). Denimal et al. reported that despite normal HDL cholesterol levels in T1DMpatients, S1P content in HDL subfractions was significantly reduced, with HDL-associated S1P demonstrating impaired functionality (24, 25). Similarly, Frej et al. demonstrated that although total plasma ApoM and S1P levels were unchanged in T1DMpatients, the distribution of ApoM/S1P complexes shifted from dense to light HDL particles, and these altered complexes exhibited markedly reduced capacity to activate S1P1 receptor downstream signaling (26, 27). Consistent with these reports, our data further support that the reduction of functional HDL-S1P in T1DM is not caused by decreased ApoM expression, but may be attributed to hyperglycemia-induced HDL structural glycation, Glycation of HDL components, as demonstrated by Tozuka et al., can impair HDL’s capacity to bind S1P and accelerate S1P degradation, providing a mechanistic link between hyperglycemia and S1P dysfunction (28, 29). Emerging evidence suggests that beyond carrier functions, the S1P axis regulates mitochondrial biology. As reviewed by Duan et al., changes in S1P production, transport, and signaling influence mitochondrial biogenesis, respiratory function, and mitophagy, thereby linking S1P dysregulation to metabolic dysfunction in diabetes. This adds a mitochondrial dimension to our understanding of the disease (29). Furthermore, a recent study by Croyal et al. in type 2 diabetes demonstrated that low HDL-S1P content was associated with coronary artery calcification and impaired anti-inflammatory properties, underscoring the clinical relevance of S1P carrier distribution (30). These findings collectively suggest that altered S1P carrier distribution rather than total S1P level alone dominates S1P functional abnormality in T1DM.

### 4.2 Biological implications of S1PR1 downregulation in intestinal T cells: post−migration events and receptor desensitization

Dynamic downregulation of S1PR1 in intestinal T cells was observed in both T1DM patients and 12-week NOD mice, implying phenotypic remodeling of intestinal T cells during disease progression. Relevant literature has reported that altered S1PR1 expression is closely related to T cell activation and migratory capacity, and impaired S1P-dependent migration exists in mature T cell subsets under autoimmune conditions (9, 31).

In this study, further Gene Ontology (GO) and KEGG enrichment analysis of CD8Teff and Treg cells from human and mouse samples also revealed significant changes in GPCR signaling, chemokine activity, cytotoxic function and cellular stress-related pathways (**Supplementary Figures 1**-**4**). These transcriptomic alterations further imply that S1PR1 remodeling is accompanied by functional reprogramming of intestinal T cells. Considering the current descriptive transcriptomic data, we cautiously consider that decreased intestinal S1PR1 expression may be related to T cell functional adaptation and microenvironmental changes during T1DM progression. Definitive inference regarding gut-to-pancreas migration and post-migration molecular imprint cannot be made without *in vitro* functional verification and *in vivo* cell tracing.

### 4.3 Association of S1P with clinical parameters and implications

Our clinical data revealed significant positive correlations between serum S1P and both HbA1c and IL−6. Kurano et al. provided a mechanistic framework, demonstrating that ApoM/S1P enhances insulin sensitivity via Akt and AMPK activation and upregulation of SIRT1, improving mitochondrial function^27^. In T1DM, this protective axis may be compromised due to S1P dysfunction, potentially explaining the positive HbA1c–S1P correlation as a marker of insufficient compensation.

The observed 22% increase in T1DM odds per 100 nmol/L S1P increment carries clinical significance. Integrating prior findings, elevated total serum S1P may reflect a compensatory response to functional S1P deficiency, with high levels indicating disease activity or tissue damage. This aligns with the association between S1P and prior hypoglycemic episodes—hypoglycemia reflects glycemic instability, during which S1P may rise as a stress−responsive molecule.

### 4.4 Limitations and future directions

Several limitations of this study should be acknowledged.

First, single-cell transcriptomic analysis and functional enrichment results remain descriptive, and we did not perform *in vitro* migration or *in vivo* cell tracing experiments to further verify the regulatory effect of the S1P-S1PR1 axis on intestinal T cell migration. Second, the clinical cohort was cross-sectional, which cannot determine the causal relationship between S1P alteration and T1DM onset and progression. Third, although we supplemented the detection of HDL-S1P and ApoM, we did not further isolate different HDL subfractions to analyze S1P distribution and functional differences. Fourth, only two representative time points of NOD mice were selected, which may not fully reflect the continuous dynamic changes of the S1P-S1PR1 axis throughout the disease course. Fifth, due to clinical sample limitations and ethical constraints, matched intestinal and pancreatic tissues from the same individual were unavailable, and direct evidence for intestinal-origin T cells infiltrating the pancreas could not be provided.

Future work will focus on *in vitro* functional validation, longer-term dynamic animal observation, and larger longitudinal clinical cohorts to further clarify the role of the S1P-S1PR1 axis in intestinal-pancreatic immune crosstalk.

## 5 Conclusions

In summary, this study characterizes S1P-S1PR1 axis alterations in T1DM based on human single-cell transcriptomics, NOD mouse model and clinical cohort data. Intestinal T cells, particularly CD8^+^ effector T cells, exhibited decreased S1PR1 expression in T1DM, with similar dynamic changes observed in NOD mice. Circulating total S1P was elevated in T1DM patients, accompanied by reduced HDL-bound S1P and stable ApoM levels, indicating functional dissociation of S1P carrier distribution. Transcriptomic enrichment further suggested functional reprogramming of intestinal CD8Teff and Treg cells. Serum S1P was independently associated with T1DM risk and correlated with glycemic and inflammatory indicators. These findings provide descriptive evidence for S1P/S1PR1 axis dysregulation and its potential involvement in intestinal immune homeostasis in T1DM, and offer a theoretical basis for further exploring S1P-related biomarker and therapeutic research.

## Data Availability

The data presented in the study are included in the article and **Supplementary Material**. Raw data are not publicly available due to ethical restrictions and patient privacy requirements but are available from the corresponding author upon reasonable request.

## Supplementary material

Supplementary Figure 1 Transcriptomic and functional enrichment of total intestinal T cells in T1DM patients versus healthy controls:**(A)** Heatmap of representative differentially expressed genes (DEGs). **(B)** Bubble plot for KEGG enrichment of downregulated DEGs. **(C)** Bubble plot for GOALL enrichment of downregulated DEGs. **(D)** Bubble plot for KEGG enrichment of upregulated DEGs. **(E)** Bubble plot for GOALL enrichment of upregulated DEGs. Bubble size represents gene count; color indicates adjusted P-value; X-axis represents GeneRatio. All enrichment plots show representative results from n = 2 independent biological replicates.

Supplementary Figure 2 Functional enrichment of intestinal CD8Teff cells in T1DM patients versus healthy controls:**(A)** Heatmap of representative DEGs. **(B)** Bubble plot for KEGG enrichment of downregulated DEGs. **(C)** Bubble plot for KEGG enrichment of upregulated DEGs. **(D)** Bubble plot for GOALL enrichment of upregulated DEGs. All enrichment plots show representative results from n = 2 independent biological replicates.

Supplementary Figure 3 Transcriptomic and functional enrichment of intestinal Treg cells between NOD4 and NOD12 mice:**(A)** Heatmap of representative DEGs. **(B)** Bubble plot for KEGG enrichment of downregulated DEGs. **(C)** Bubble plot for GOALL enrichment of downregulated DEGs. **(D)** Bubble plot for GOALL enrichment of upregulated DEGs. **(E)** Bubble plot for KEGG enrichment of upregulated DEGs. All enrichment plots show representative results from n = 2 independent biological replicates.

Supplementary Figure 4 Transcriptomic and functional enrichment of intestinal CD8Teff cells between NOD4 and NOD12 mice:**(A)** Heatmap of representative DEGs. **(B)** Bubble plot for KEGG enrichment of downregulated DEGs. **(C)** Bubble plot for GOALL enrichment of downregulated DEGs. **(D)** Bubble plot for GOALL enrichment of upregulated DEGs. **(E)** Bubble plot for KEGG enrichment of upregulated DEGs. All enrichment plots show representative results from n = 2 independent biological replicates.

## Glossary

ApoM: Apolipoprotein M
BMI: Body mass index
CON: Healthy control
CD8Teff: CD8⁺ effector T cell
Treg: Regulatory T cell
DEG: Differentially expressed gene
ELISA: Enzyme-linked immunosorbent assay
GADA: Glutamic acid decarboxylase antibody
GO: Gene Ontology
GSEA: Gene Set Enrichment Analysis
HbA1c: Glycated hemoglobin
HC: Healthy control
HDL: High-density lipoprotein
HDL-S1P: High-density lipoprotein-bound sphingosine-1-phosphate
hs-CRP: High-sensitivity C-reactive protein
IAA: Insulin autoantibody
IL-6: Interleukin-6
IQR: Interquartile range
KEGG: Kyoto Encyclopedia of Genes and Genomes
LDL-C: Low-density lipoprotein cholesterol
MBTPS1: Membrane-bound transcription factor peptidase, site 1
MoCA: Montreal Cognitive Assessment
MMSE: Mini-Mental State Examination
NOD: Non-obese diabetic
OH: Orthostatic hypotension
PSQI: Pittsburgh Sleep Quality Index
S1P: Sphingosine-1-phosphate
S1PR1–S1PR5: Sphingosine-1-phosphate receptor 1–5
scRNA-seq: Single-cell RNA sequencing
SPF: Specific pathogen-free
T1DM: Type 1 diabetes mellitus
TC: Total cholesterol
TG: Triglycerides
UACR: Urine albumin-to-creatinine ratio
UMAP: Uniform manifold approximation and projection
HAMD-17: 17-item Hamilton Depression Rating Scale
GPCR: G protein-coupled receptor.
