# Supplementary material for: Alterations of sphingosine-1-phosphate and its receptors in type 1 diabetes mellitus: an integrated clinical and single-cell transcriptomic study
Source: Front Immunol. 2026 Jun 8;17:1838952. doi: 10.3389/fimmu.2026.1838952 (PMC13284128; doi:10.3389/fimmu.2026.1838952)
Supplement: Supplementary file 1 [file Table1.docx]

Single-Cell RNA Sequencing and Bioinformatic Analysis

**Con and T1DM**

**Quality control, dimension-reduction and clustering (Scanpy)**

Scanpy v1.8.1 was used for quality control, dimensionality reduction and clustering under Python 3.7. For each sample dataset, we filtered expression matrix by the following criteria: 1) cells with gene count less than 200 or with top 2% gene count were excluded; 2) cells with top 2% UMI count were excluded; 3) cells with mitochondrial content $>$ 50% were excluded; 4) genes expressed in less than 5 cells were excluded. After filtering, 22232 cells were retained for the downstream analyses, with on average 634 genes and 1915 UMIs per cell. The raw count matrix was normalized by total counts per cell and logarithmically transformed into normalized data matrix. Top 2000 variable genes were selected by setting flavor = ‘seurat’. Principle Component Analysis (PCA) was performed on the scaled variable gene matrix, and top 20 principle components were used for clustering and dimensional reduction. Cells were separated into 18 clusters by using Louvain algorithm and setting resolution parameter at 1.2. Cell clusters were visualized by using Uniform Manifold Approximation and Projection (UMAP).

Reference：

Wolf, F., Angerer, P. & Theis, F. SCANPY: large-scale single-cell gene expression data analysis. Genome Biol 19, 15 (2018). <https://doi.org/10.1186/s13059-017-1382-0>

**Batch Effect removal**

Harmony: Batch effect between samples was removed by Harmony v1.0 using the top 20 principal components from PCA.

Reference：

Butler, A., Hoffman, P., Smibert, P. et al. Integrating single-cell transcriptomic data across different conditions, technologies, and species. Nat Biotechnol 36, 411–420 (2018). <https://doi.org/10.1038/nbt.4096>

**Cell type annotation**

**Cell-type recognition with Cell-ID**

Cell-ID is multivariate approach that extracts gene signatures for each individual cell and perform cell identity recognition using hypergeometric tests (HGT). Dimensionality reduction was performed on normalized gene expression matrix through multiple correspondence analysis, where both cells and genes were projected in the same low dimensional space. Then a gene ranking was calculated for each cell to obtain most featured gene sets of that cell. HGT were performed on these gene sets against brain reference from SynEcoSys database, which contains all cell-type's featured genes. Identity of each cell was determined as the cell-type has the minimal HGT p value. For cluster annotation, Frequency of each cell-type was calculated in each cluster, and cell-type with highest frequency was chosen as cluster’s identity.

Reference：

1. Cortal, A., Martignetti, L., Six, E. & Rausell, A. Gene signature extraction and cell identity recognition at the single-cell level with Cell-ID. Nature Biotechnology 1–8 (2021) doi:10.1038/s41587-021-00896-6.
2. Yan Zhang, Bingyu Li, Jiachen Duan, Xuezhen Chen, Xiaogang Zhang, Jun Ye, Ana Veloso, Jue Fan, Nan Fang. Preprint at bioRxiv https://doi.org/10.1101/2023.02.14.528566

The cell type identification of each cluster was determined according to the expression of canonical markers from the reference database SynEcoSys^TM^ (Singleron Biotechnology). SynEcoSys^TM^ contains collections of canonical cell type markers for single-cell seq data, from CellMakerDB, PanglaoDB and recently published literatures. The canonical markers and their corresponding cell types were listed in Table x.

**Subtyping of major cell types**

To obtain a high-resolution map of XXX, cells from the specific cluster were extracted and reclustered for more detailed analysis following the same procedures described above.

Reference：

Yan Zhang, Bingyu Li, Jiachen Duan, Xuezhen Chen, Xiaogang Zhang, Jun Ye, Ana Veloso, Jue Fan, Nan Fang. Preprint at bioRxiv https://doi.org/10.1101/2023.02.14.528566

**Filtering Cell Doublets**

Cell doublets were estimated based on the expression pattern of canonical cell markers. Any clusters enriched with multiple cell type-specific markers were excluded for downstream analysis.

**NOD4 NOD12**

**Quality control, dimension-reduction and clustering (Scanpy)**

Scanpy v1.9.3was used for quality control, dimensionality reduction and clustering under Python 3.10. For each sample dataset, we filtered expression matrix by the following criteria: 1) cells with gene count less than 200 or with top 2% gene count were excluded; 2) cells with top 2% UMI count were excluded; 3) cells with mitochondrial content $>$ 50% were excluded; 4) genes expressed in less than 5 cells were excluded. After filtering, 40373 cells were retained for the downstream analyses, with on average 1558 genes and 5677 UMIs per cell. The raw count matrix was normalized by total counts per cell and logarithmically transformed into normalized data matrix. Top 2000 variable genes were selected by setting flavor = ‘seurat_v3’. Principle Component Analysis (PCA) was performed on the scaled variable gene matrix, and top 20 principle components were used for clustering and dimensional reduction. Cells were separated into 25 clusters by using Louvain algorithm and setting resolution parameter at 1.2. Cell clusters were visualized by using Uniform Manifold Approximation and Projection (UMAP). Batch effect between samples was removed by Harmony v1.0 using the top 20 principal components from PCA.

Reference：

1. Wolf, F., Angerer, P. & Theis, F. SCANPY: large-scale single-cell gene expression data analysis. Genome Biol 19, 15 (2018). <https://doi.org/10.1186/s13059-017-1382-0>
2. Butler, A., Hoffman, P., Smibert, P. et al. Integrating single-cell transcriptomic data across different conditions, technologies, and species. Nat Biotechnol 36, 411–420 (2018). <https://doi.org/10.1038/nbt.4096>

**Celltype annotation**

The cell type identification of each cluster was determined according to the expression of canonical markers from the reference database SynEcoSys^TM^ (Singleron Biotechnology). SynEcoSys^TM^ contains collections of canonical cell type markers for single-cell seq data, from CellMakerDB, PanglaoDB and recently published literatures.

Reference：

Yan Zhang, Bingyu Li, Jiachen Duan, Xuezhen Chen, Xiaogang Zhang, Jun Ye, Ana Veloso, Jue Fan, Nan Fang. Preprint at bioRxiv https://doi.org/10.1101/2023.02.14.528566

**Differentially expressed genes (DEGs) analysis (scanpy)**

To identify differentially expressed genes (DEGs), we used the scanpy.tl.rank_genes_groups() function based on Wilcoxon rank sum test with default parameters, and selected the genes expressed in more than 10% of the cells in either of the compared groups of cells and with an average log(Fold Change) value greater than 0.25 as DEGs. Adjusted p value was calculated by benjamini-hochberg correction and the value 0.05 was used as the criterion to evaluate the statistical significance.

**Pathway enrichment analysis**

To investigate the potential functions of xxx, Gene Ontology (GO) and Kyoto Encyclopedia of Genes and Genomes (KEGG) analysis were used with the “clusterProfiler” R package v 3.16.1 (ref1). Pathways with p_adj value less than 0.05 were considered as significantly enriched. Selected significant pathways were plotted as bar plots. Gene Ontology gene sets including molecular function (MF), biological process (BP), and cellular component (CC) categories were used as reference.

Reference

Yu G, Wang LG, Han Y, He QY. clusterProfiler: an R package for comparing biological themes among gene clusters. Omics a J Integr Biol. 2012;16(5):284–7.
