# Supplementary figures and images for: Alterations of sphingosine-1-phosphate and its receptors in type 1 diabetes mellitus: an integrated clinical and single-cell transcriptomic study
Source: Front Immunol. 2026 Jun 8;17:1838952. doi: 10.3389/fimmu.2026.1838952 (PMC13284128; doi:10.3389/fimmu.2026.1838952)

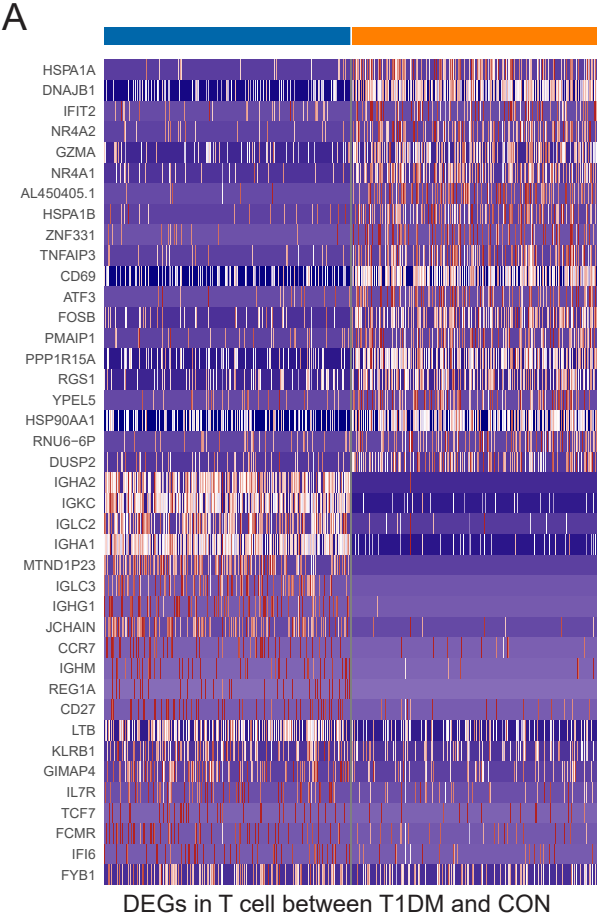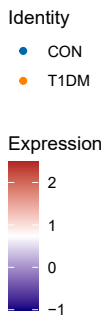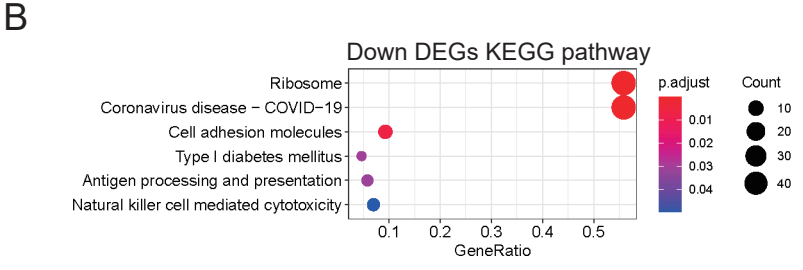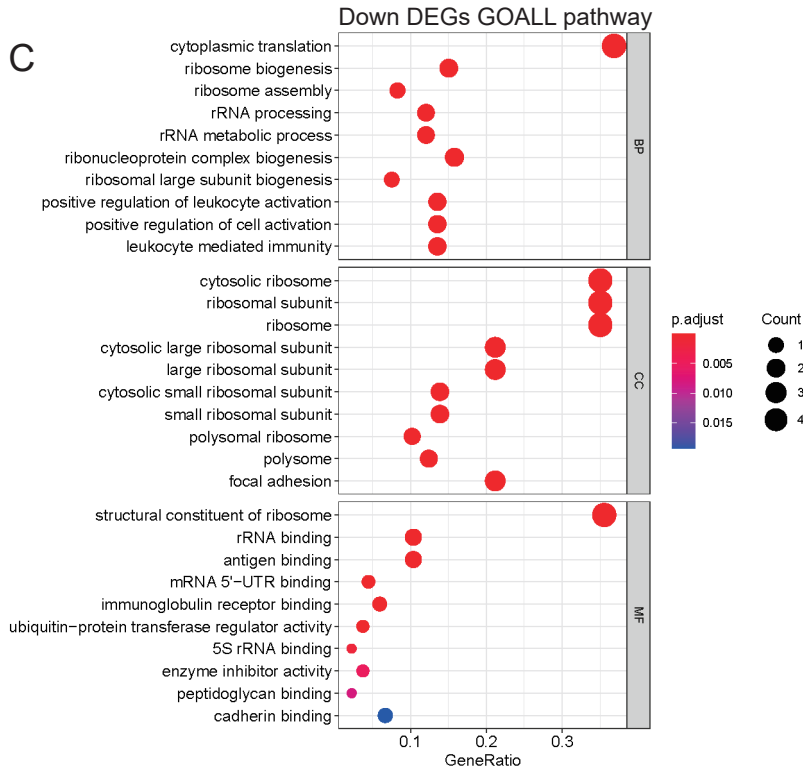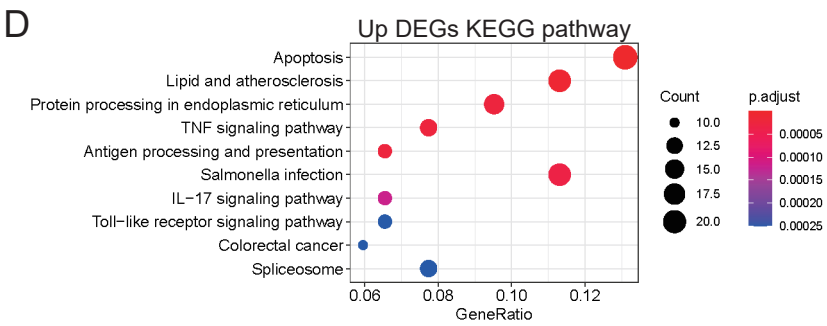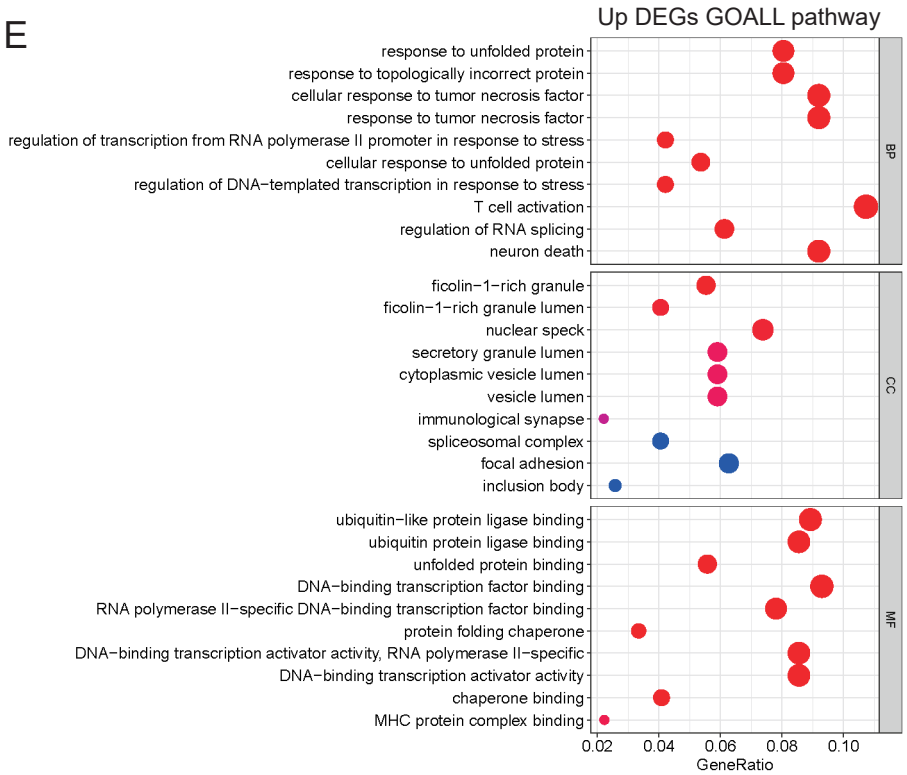

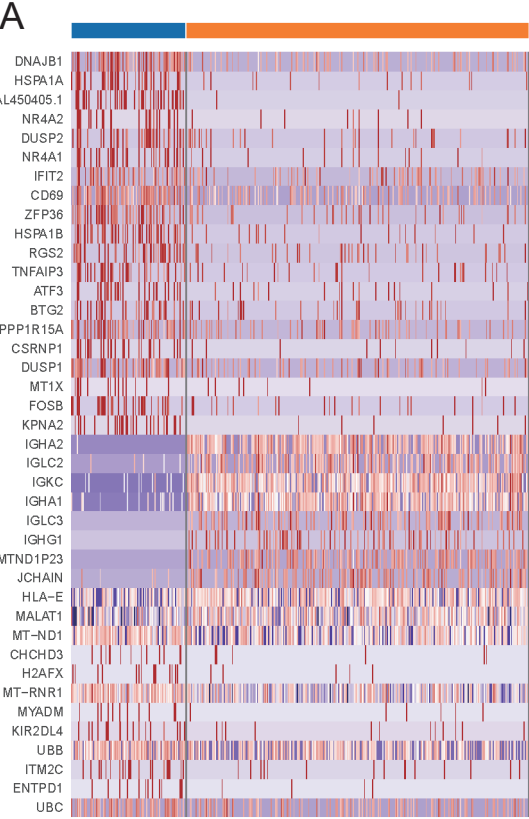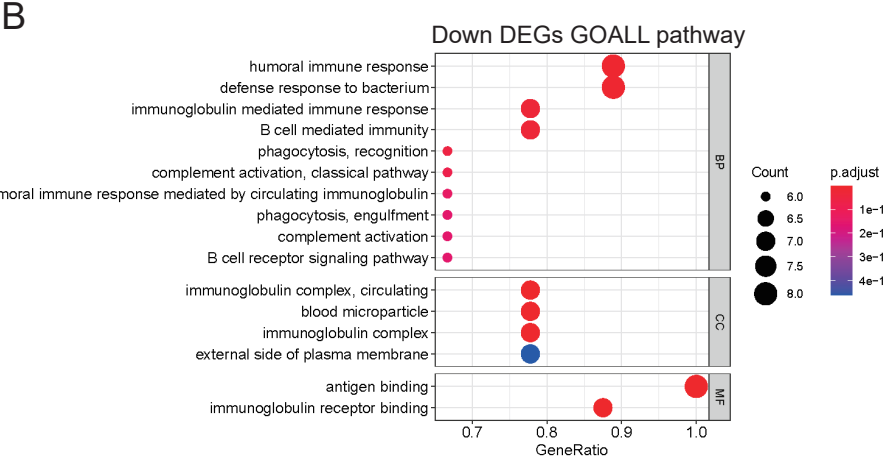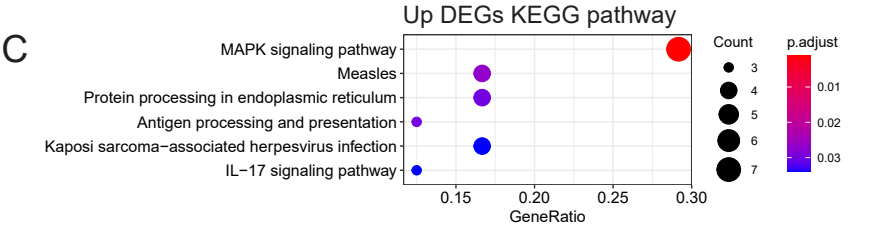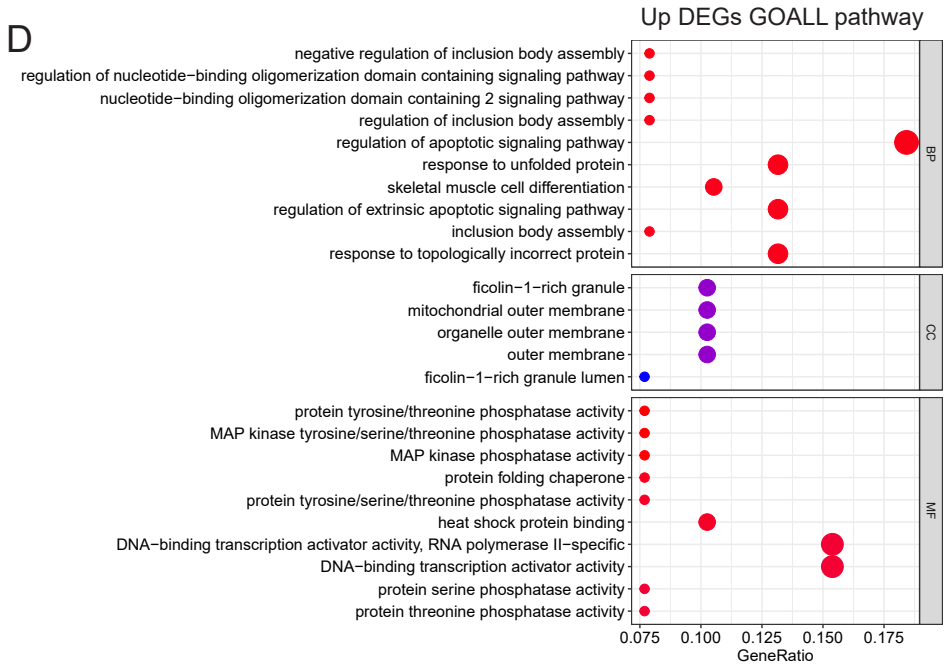

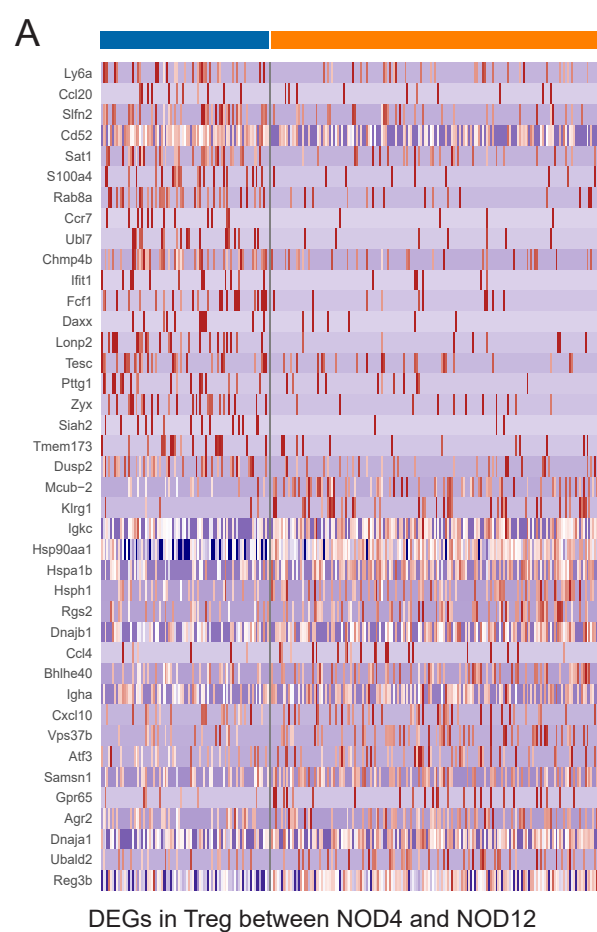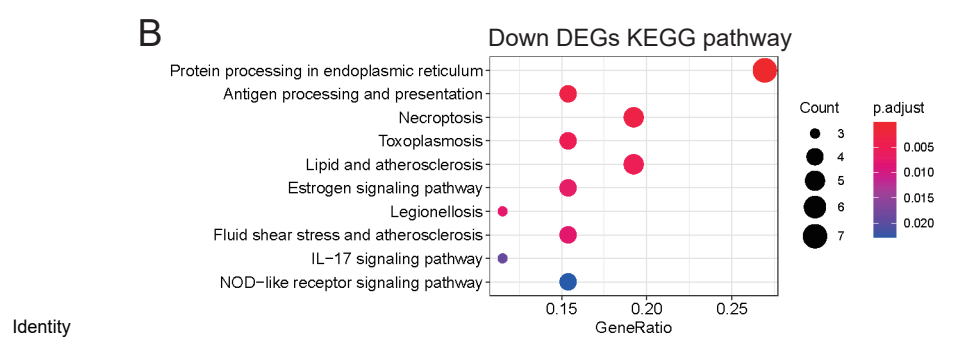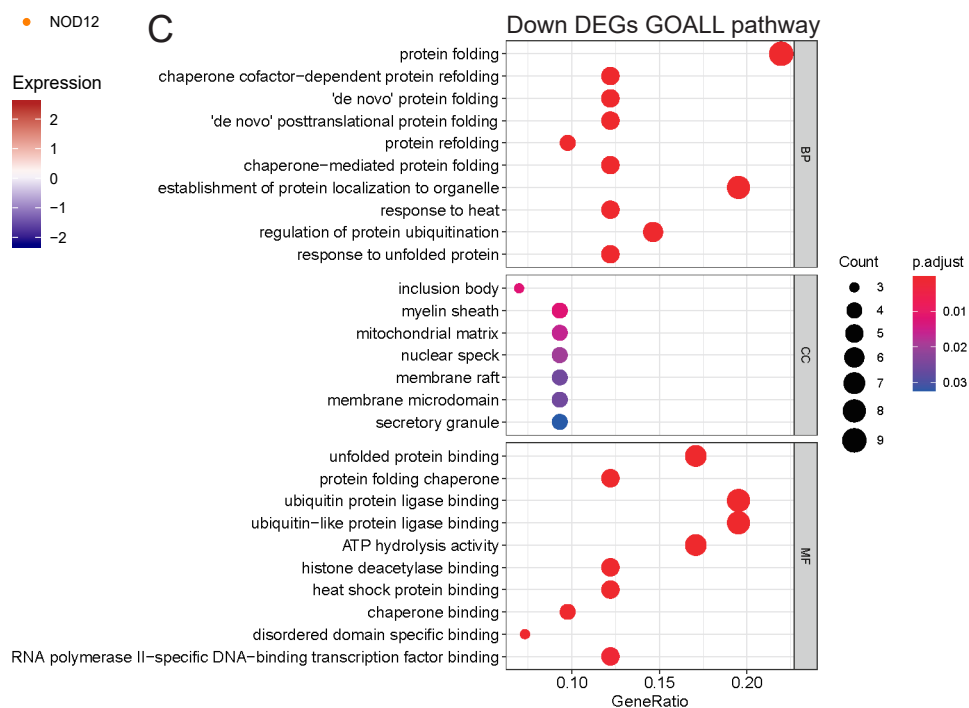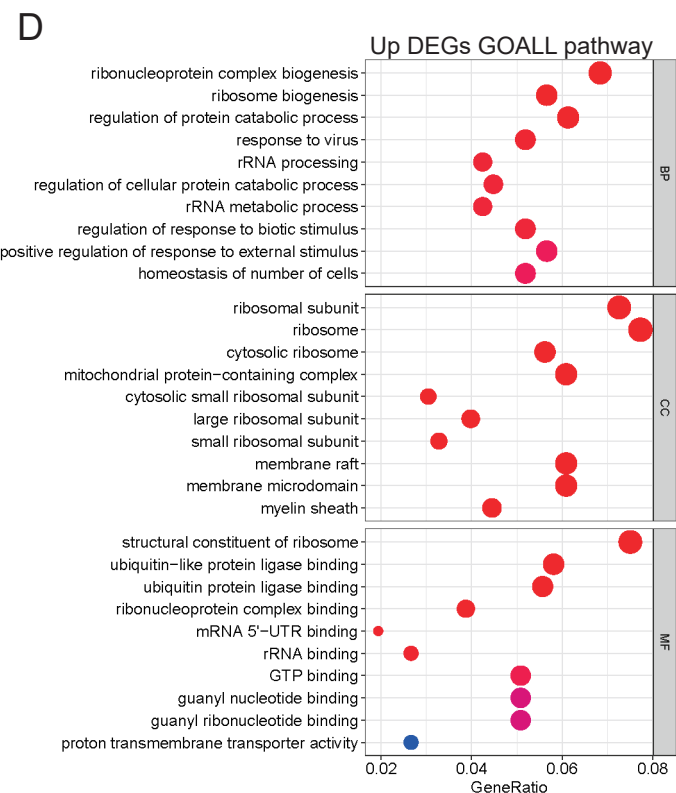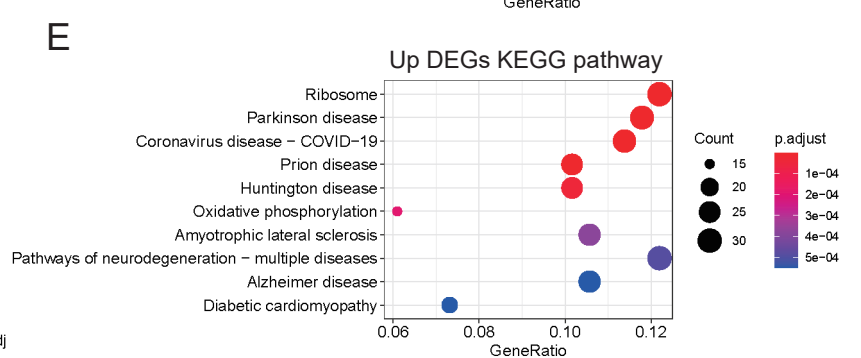

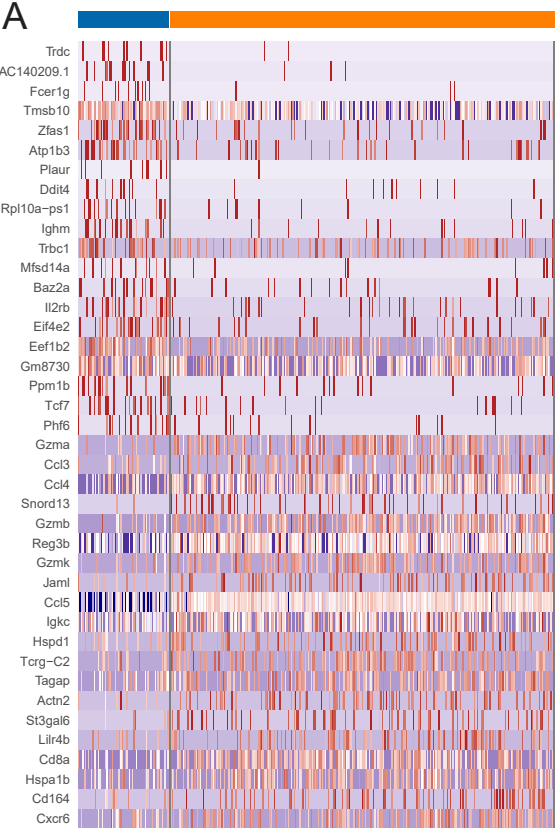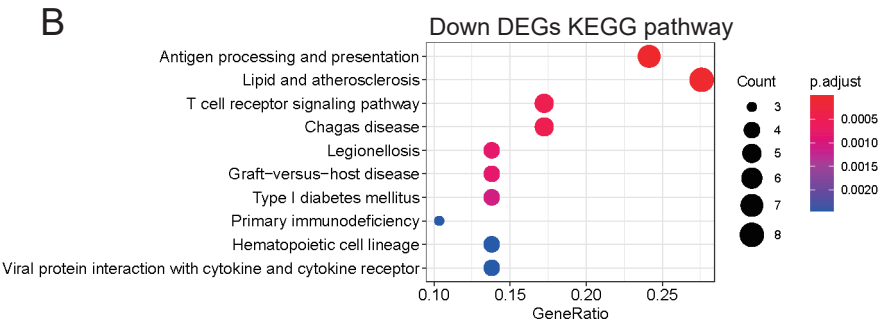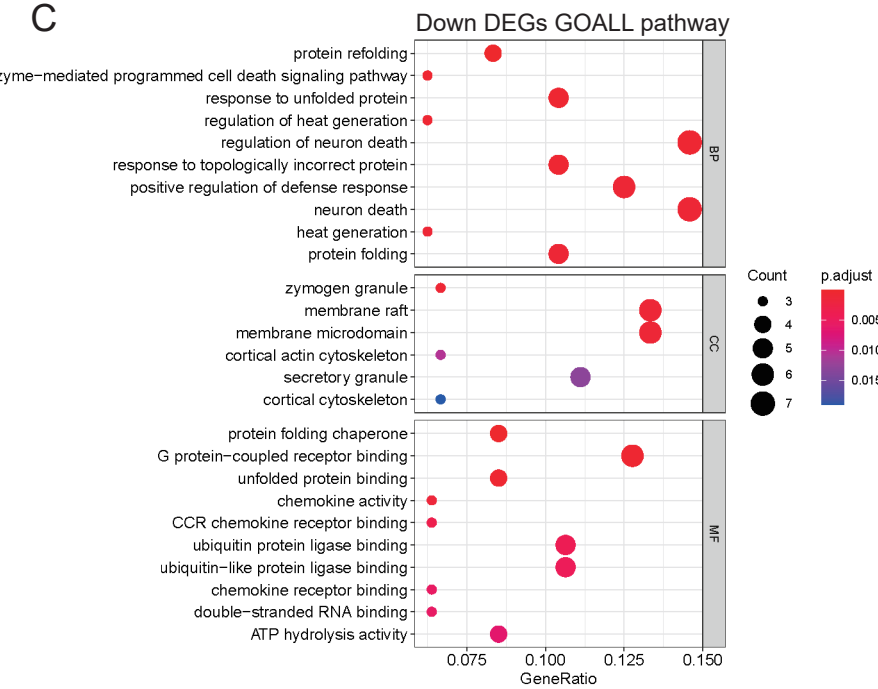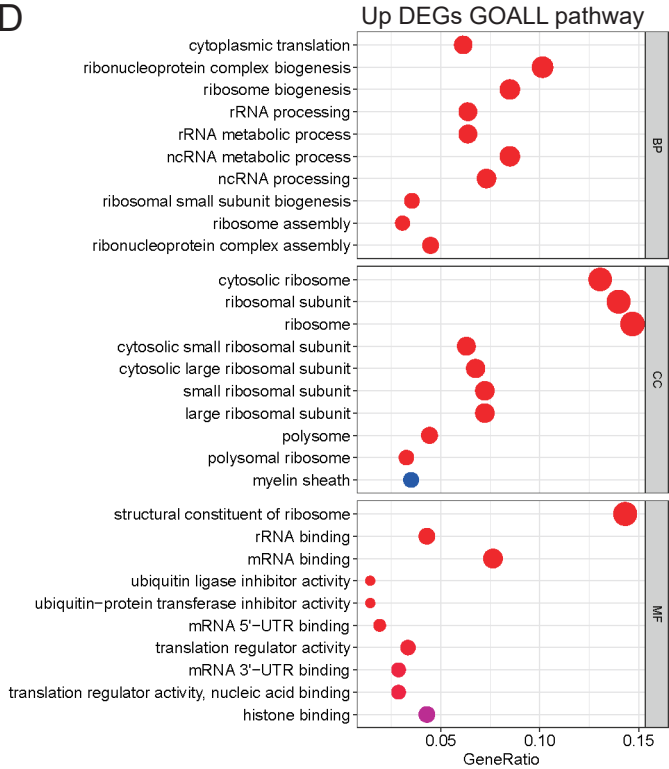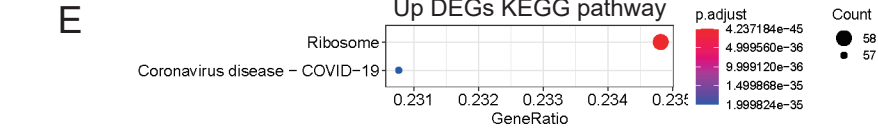

Supplement: Supplementary Figure 1 — Transcriptomic and functional enrichment of total intestinal T cells in T1DM patients versus healthy controls:(A) Heatmap of representative differentially expressed genes (DEGs). (B) Bubble plot for KEGG enrichment of downregulated DEGs. (C) Bubble plot for GOALL enrichment of downregulated DEGs. (D) Bubble plot for KEGG enrichment of upregulated DEGs. (E) Bubble plot for GOALL enrichment of upregulated DEGs. Bubble size represents gene count; color indicates adjusted P-value; X-axis represents GeneRatio. All enrichment plots show representative results from n = 2 independent biological replicates. [file DataSheet1.pdf]
